# The Cult of the Sun-Bath

**Published:** 1912-08-10

**Authors:** Dudley D'A. Wright


### THE EDITOR'S LETTER-BOX

### "The Cult of the Sun-Bath."

*To the Editor of* The Hospital.

Sir,—In your issue of July 20 there is a paragraph under the above heading in which you rightly say that in this country "the regular sun-bather is at a disadvantage as compared with the season-ticket holder at a Turkish bath establishment," and also that "not a great deal has been heard in England of the therapeutic value of the sun-bath."

There is happily, however, in England a growing tendency to recognise the efficacy of solar therapeutics, not only for tuberculous disease, but for other maladies; and it may not be known that there are now several institutions within a radius of fifty miles of London where the sun- and air-bath play a prominent part in treatment, and at one of these, in Hampshire, sun-bathing is successfully carried out in the winter months when a minimum of sunlight visits us.

Those who have once tried these methods soon become convinced of their utility, and practice them whenever occasion arises; and it is highly desirable that a more extended knowledge of the value of the sun and air baths be spread abroad in this country and suitable opportunities be made for indulging in the same.—I am, Sir, yours, etc.,

Dudley D'A. Wright, F.R.C.S.

